# A Novel Risk-Prediction Scoring System for Sepsis among Patients with Acute Pancreatitis: A Retrospective Analysis of a Large Clinical Database

**DOI:** 10.1155/2022/5435656

**Published:** 2022-02-22

**Authors:** Aozi Feng, Xi Ao, Ning Zhou, Tao Huang, Li Li, Mengnan Zeng, Jun Lyu

**Affiliations:** ^1^Department of Clinical Research, The First Affiliated Hospital of Jinan University, Guangzhou, Guangdong 510632, China; ^2^The Science & Education Office, The First Affiliated Hospital, Jinan University, Guangzhou, Guangdong 510632, China; ^3^College of Pharmacy, Henan University of Chinese Medicine, Zhengzhou, Henan 450046, China

## Abstract

**Background:**

The prognosis is poor when acute pancreatitis (AP) progresses to sepsis; therefore, it is necessary to accurately predict the probability of sepsis and develop a personalized treatment plan to reduce the disease burden of AP patients.

**Methods:**

A total of 1295 patients with AP and 43 variables were extracted from the Medical Information Mart for Intensive Care (MIMIC) IV database. The included patients were randomly assigned to the training set and to the validation set at a ratio of 7 : 3. The chi-square test or Fisher's exact test was used to test the distribution of categorical variables, and Student's *t*-test was used for continuous variables. Multivariate logistic regression was used to establish a prognostic model for predicting the occurrence of sepsis in AP patients. The indicators to verify the overall performance of the model included the area under the receiver operating characteristic curve (AUC), calibration curves, the net reclassification improvement (NRI), the integrated discrimination improvement (IDI), and a decision curve analysis (DCA).

**Results:**

The multifactor analysis results showed that temperature, phosphate, calcium, lactate, the mean blood pressure (MBP), urinary output, Glasgow Coma Scale (GCS), Charlson Comorbidity Index (CCI), sodium, platelet count, and albumin were independent risk factors. All of the indicators proved that the prediction performance and clinical profitability of the newly established nomogram were better than those of other common indicators (including SIRS, BISAP, SOFA, and qSOFA).

**Conclusions:**

The new risk-prediction system that was established in this research can accurately predict the probability of sepsis in patients with acute pancreatitis, and this helps clinicians formulate personalized treatment plans for patients. The new model can reduce the disease burden of patients and can contribute to the reasonable allocation of medical resources, which is significant for tertiary prevention.

## 1. Background

Pancreatitis is the main cause of hospitalization for patients with gastrointestinal-related diseases, and it is associated with considerable morbidity, mortality, and socioeconomic burdens [1]. Acute pancreatitis (AP) is one of the most common causes of hospitalization for patients with gastrointestinal diseases in the United States [2] and is also one of the main causes of death in hospitals [3]. AP is an inflammatory disease of the pancreas. The cause of AP has been discovered, but its pathogenesis is still controversial. Most researchers believe that when the intracellular protective mechanisms that prevent trypsinogen activation or reduce trypsin activity are overwhelmed, AP will occur [4]. The incidence of AP is 13∼45 per 100,000 persons [5, 6]. In 2009, AP caused approximately 275,000 hospitalizations [7] (it has more than doubled since 1988 [8]). The incidence of chronic pancreatitis (CP) is 5-12/100,000 persons [9, 10]; the prevalence of CP is approximately 50/100,000 persons [11]. A number of population research reports have indicated that the incidences of AP and CP are still increasing, and the increase in the incidence of AP is even greater [10–12]. Due to the different social developments that have occurred in different regions, there are differences in the regional distribution of pancreatitis. Compared with the countries in Europe and America and considering the rapid urbanization and increasing affluence of China and India, the changes in the diet and the increase in alcohol consumption have led to an increased burden of pancreatitis in Asia and have caused it to be more serious [13].

Sepsis is a life-threatening systemic inflammatory response syndrome (SIRS) caused by the host's dysregulated response to infection, which ultimately leads to septic shock and multiple organ failure. Pancreatic injury is considered to be an important pathological change in sepsis [14]. AP is characterized by a strong proinflammatory response, which may lead to SIRS, organ failure, and death [15]. Among the cases of AP, the total mortality of patients with acute necrotizing pancreatitis is in the range of 10-15%, 40–70% of patients will develop secondary pancreatic infection and sepsis, and the mortality rate is 80% [16]. AP has two peak periods of death. The first peak period is due to the large number of patients who develop SIRS and subsequently develop multiple organ failure. The second peak of death is usually detected at a later time (at least two weeks after the onset of acute pancreatitis) when it is usually accompanied by infection [17]. Sepsis is the usual clinical manifestation of the patients at this time. Despite the improvements in antibiotic treatments, ventilator management, and resuscitation strategies, the prognosis of sepsis is still unsatisfactory due to the insufficient awareness of sepsis, the delays in evaluation, and development of antibiotic resistance [18, 19]. At the same time, it is difficult for clinicians to distinguish between patients with benign pancreatitis and patients with severe pancreatitis [20]. Therefore, it is necessary to effectively identify AP at an early stage to avoid further deterioration of the patient and the development of sepsis. The current tools for predicting the severity of severe-related diseases include the SIRS standards, SOFA, and qSOFA scores [21, 22], including BISAP, an independent predictor of adverse pancreatitis outcomes [23]. However, these tools are general-purpose and have limitations in predicting specific diseases. Askim Å et al. pointed out that the qSOFA score does not perform well in predicting sepsis and death [24].

This article aims to explore the risk factors that cause sepsis in patients with AP, establish an independent predictive tool-nomogram, predict the possibility of sepsis in AP patients, and provide a reference for medical personnel to formulate a personalized treatment plan. It has a strong tertiary prevention meaning.

## 2. Materials and Methods

### 2.1. Data Source

We conducted a retrospective cohort study with data extracted from the Medical Information Mart for Intensive Care, version 1.0 (MIMIC-IV v1.0) database. The MIMIC-IV is a large, single-center and open-access database that includes 524,740 admissions for 382,278 patients admitted to the intensive care unit at Beth Israel Deaconess Medical Center in Boston from 2008 to 2019 [25]. We completed online courses and exams to gain access to the database (record ID: 38455175). Because the MIMIC-IV database is approved by the Institutional Review Boards of Beth Israel Deaconess Medical Center and Massachusetts Institute of Technology and because all patient information in the database is anonymous, informed consent was not required for use of the MIMIC-IV database for this study.

### 2.2. Case Screening and Variable Selection

**Figure 1:**
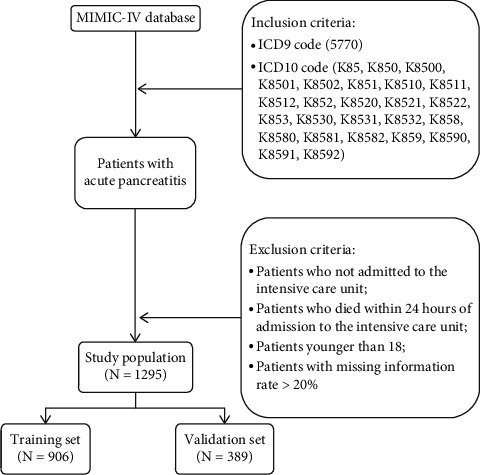
Flowchart of study cohort selection.

We used Navicat Premium (version 11.2.7.0) to run the structured query language to extract data from the MIMIC-IV database. Cases of acute pancreatitis (AP) were identified by the International Classification of Diseases 9th edition (ICD-9) code 5770 and the International Classification of Diseases 10th edition (ICD-10) code K85. Patients who were not admitted to the intensive care unit (ICU), who died within 24 hours of admission to the ICU, whose age was less than 18 years old, or whose missing information ratio was greater than 20%, were excluded. The flowchart of the inclusion and exclusion criteria is shown in Figure 1.

The outcome of this study was the occurrence of sepsis. Sepsis was defined according to the Sepsis 3.0 criteria, that is, a suspected or confirmed infection plus an acute increase of more than two points in the Sequential Organ Failure Assessment (SOFA) score. We extracted the following potential prognostic indicators: (a) demographic data: age, sex, ethnicity (white, black, and other), weight, and pleural effusion (PE); (b) mean value of the vital signs within 24 hours after admission to the ICU: heart rate (HR), mean blood pressure (MBP), respiratory rate (RR), temperature, 24-hour urine output, and percutaneous oxygen saturation (SpO2); (c) the initial values of laboratory examination indicators after admission to the ICU: anion gap (AG), sodium, potassium, magnesium, calcium, chloride, phosphate, bicarbonate, white blood cell (WBC) count, red blood cell (RBC) count, hematocrit, mean corpuscular hemoglobin concentration (MCHC), RBC distribution width (RDW), international normalized ratio (INR), creatinine, blood urea nitrogen (BUN), lactate, albumin, bilirubin, alkaline phosphatase (ALP), aspartate transaminase (AST), alanine transaminase (ALT), glucose, lipase, and lactate dehydrogenase (LD); D) disease severity: CCI alkaline phosphatase (ALP), aspartate transaminase (AST), alanine transaminase (ALT).

Common prognostic scoring systems for patients with acute pancreatitis, such as blood urea nitrogen, impaired mental status, systemic inflammatory response syndrome, age and pleural effusion (BISAP), SOFA, qSOFA, and systemic inflammatory response syndrome (SIRS), were calculated using the corresponding criteria.

### 2.3. Statistical Analysis

Multiple imputation was adopted for any missing values of the variables. To reduce the risk for an information bias, only variables whose missing ratio was less than 20% were included in this study. The final included cases were randomly assigned to the training set and the validation set at a ratio of 7 : 3. Categorical variables were described as frequencies (percentages), and differences between the two sets were compared by the chi-square test or Fisher's exact test as appropriate. Continuous variables were first tested by the Shapiro-Wilk test to assess whether they conformed to a normal distribution. For those variables that conformed to a normal distribution, the mean and standard deviation were used to describe these variables, and Student's *t*-test was used to test the differences. Those variables that did not conform to a normal distribution were described as medians (interquartile-range values), and the Mann–Whitney *U*-test was used to analyze the differences between the groups.

In the training set, multivariate logistic regression was used to establish a prognostic model for predicting the occurrence of sepsis in AP patients. A backward stepwise method was used to identify independent prognostic factors, and the probability thresholds for entry and removal were 0.05 and 0.10, respectively. The odds ratio (OR) and 95% confidence interval (CI) for each independent prognostic factor were calculated. For continuous variables in the final model that contained all of the independent prognostic factors, the variance inflation factor (VIF) was used to test whether multicollinearity existed between the prognostic factors (an arithmetic square root of VIF > 2 was considered evidence of the existence of multicollinearity) [26]. The final model was presented in the form of a nomogram.

The discriminative ability, calibration degree, and clinical application value of the nomogram were validated in the training and validation sets. The indicators for assessing the discriminative ability included the area under the receiver operating characteristic curve (AUC), NRI, and integrated discrimination improvement (IDI), and their 95% CIs were estimated by bootstrapping. The receiver operating characteristic (ROC) curve is a commonly used tool to reflect the discriminative ability. After determining the optimal truncation value by Youden's index, we also determined the sensitivity and specificity of the nomogram at that value. The NRI and IDI are relatively new indicators. They reflect the improved performance of the new model compared to the old model. Compared with AUC, they are more sensitive to the difference in the discriminative abilities between two models [27]. The calibration degree was evaluated by the Hosmer-Lemeshow test and a calibration curve. A *P* value for the Hosmer-Lemeshow test >0.05 and a calibration curve close to the diagonal were considered good calibration. A DCA showed the net benefits from the clinical intervention under guidance of the prognostic models and is a tool to reflect the clinical effectiveness of the model [28].

All statistical analyses were performed by R software (version 4.0.0), and a two-sided *P* value <0.05 was regarded as statistically significant.

## 3. Results

### 3.1. Characteristics of the Patients

After the inclusion and exclusion criteria were applied, the final study cohort consisted of 1295 patients, with 906 patients assigned to the training set and 389 assigned to the validation set, and all of the characteristics were evenly distributed between the two sets. As shown in Table 1, the median age of the patients was 59 [47, 71] years, and a large proportion of the patients were male (59.2%) and white (71.8%). A minority of the patients (15.1%) had PE, and a majority of patients were conscious (the median value of the GCS was 15). Ultimately, 741 patients (57.2%) developed sepsis.

### 3.2. Nomogram Development

The independent prognostic factors that were identified by the backward stepwise method included the MBP, temperature, urinary output, the GCS, the CCI, sodium, calcium, phosphate, the platelet count, lactate, and albumin, as shown in Table 2. The MBP (OR = 0.975, 95%CI = 0.964–0.987), urine output (OR = 0.999, 95%CI = 0.998–1.000), the GCS (OR = 0.860, 95%CI = 0.763–0.969), calcium (OR = 0.841, 95%CI = 0.730–0.970), the platelet count (OR = 0.999, 95%CI = 0.998–1.000), and albumin (OR = 0.763, 95%CI = 0.609–0.957) were the protective factors, while temperature (OR = 1.876, 95%CI = 1.437–2.449), the CCI (OR = 1.098, 95%CI = 1.035–1.164), sodium (OR = 1.027, 95%CI = 1.001–1.054), phosphate (OR = 1.264, 95%CI = 1.134–1.409), and lactate (OR = 1.131.027, 95%CI = 1.039–1.053) were risk factors. The VIF was calculated, and none of the variables mentioned above had an arithmetic square root of VIF > 2, indicating that multicollinearity did not exist in the model.

**Figure 2:**
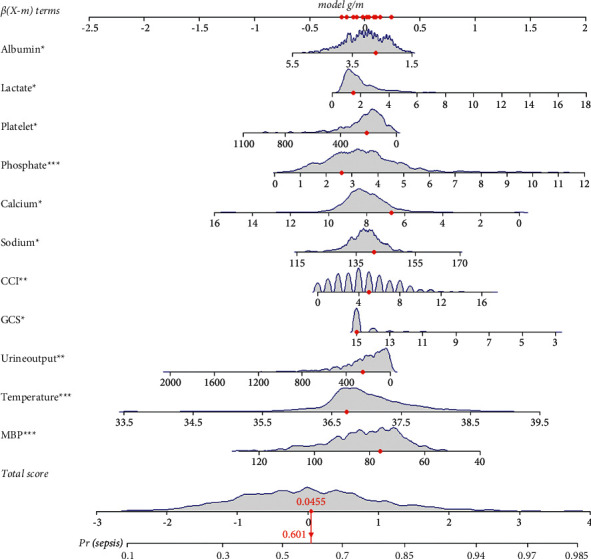
Nomogram for predicting occurrence of sepsis in patients with acute pancreatitis. Abbreviations: MBP, mean blood pressure; GCS, Glasgow Coma Scale; CCI, Charlson_comorbidity_index.

The nomogram containing all of the independent prognostic factors is shown in Figure 2. The factor that had the most prognostic value was temperature, followed by phosphate, calcium, lactate, the MBP, urinary output, the GCS, the CCI, sodium, the platelet count, and albumin. The nomogram was very convenient to use. Each characteristic of the AP patients corresponded to a risk score. We could add up all of the corresponding scores of all of the factors to obtain a total score, and the probability corresponding to the total score is the probability of that patient developing sepsis.

### 3.3. Nomogram Validation

**Figure 3:**
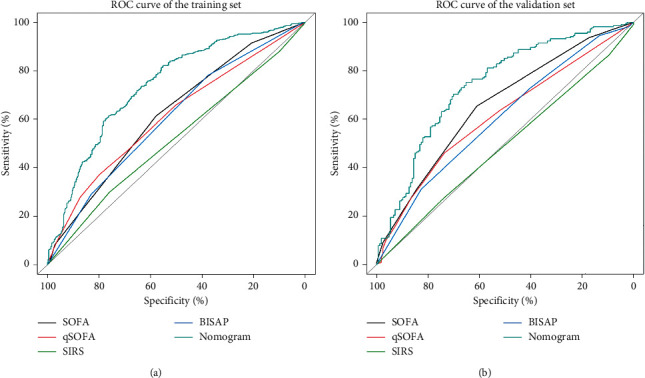
ROC curves for the nomogram and other scoring systems. (a) Training set; (b) validation set. Abbreviations: BISAP, blood urea nitrogen, impaired mental status, systemic inflammatory response syndrome, age and pleural effusion; SOFA, Sequential Organ Failure Assessment; SIRS, systemic inflammatory response syndrome.

**Figure 4:**
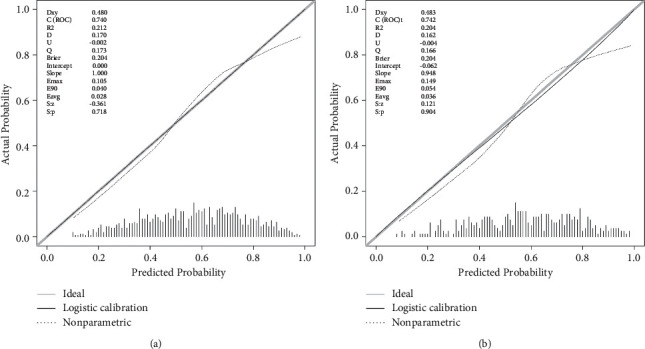
Calibration curves for the nomogram. (a) Training set; (b) validation set.

**Figure 5:**
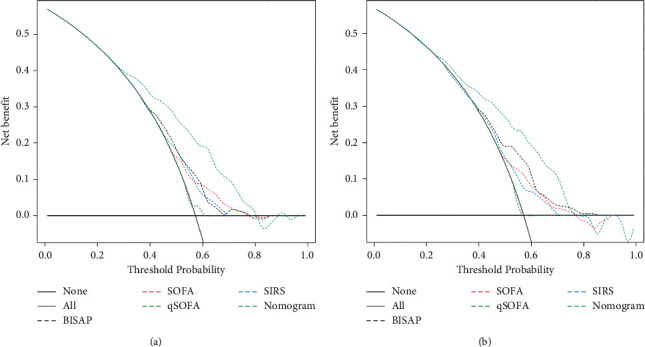
Decision curve analysis for the nomogram and other scoring systems. (a) Training set; (b) validation set. Abbreviations: BISAP, blood urea nitrogen, impaired mental status, systemic inflammatory response syndrome, age and pleural effusion; SOFA, Sequential Organ Failure Assessment; SIRS, systemic inflammatory response syndrome.

We compared the difference in the discriminative ability between the nomogram and commonly used scoring systems in predicting the occurrence of sepsis in patients with AP, and the results are shown in Table 3. The AUC values of the nomogram were 0.730 (0.700–0.765) in the training set and 0.732 (0.680–0.780) in the validation set, which were significantly higher than those of the other scoring systems. As seen from the ROC curves in Figure 3, the ROC curves of the nomogram were both higher than those of the other scoring systems in both the training and validation sets. The optimal truncation value and its corresponding sensitivity and specificity were 0.607, 76.227%, and 61.464% in the training set and 0.554, 65.269%, and 75.225% in the validation set, respectively. Both the NRI and IDI values were positive, and their corresponding *P* values were less than 0.05. All of these results indicated that the nomogram has a significantly higher degree of discriminative ability than other scoring systems. The results of the Hosmer-Lemeshow test had no statistical significance in the two data sets (in the training set: *x*_2_ = 16.790, *P*=0.052; in the validation set: *x*_2_ = 15.341, *P*=0.082), and the calibration curves in Figure 4 were both close to the diagonal, which illustrated that the nomogram had a good calibration degree. In the DCA curves in Figure 5, when the threshold probability was between 0.3 and 0.8, the curves corresponding to the nomogram were always higher than those of the other scoring systems, indicating that clinical intervention guided by the nomogram can achieve higher net benefits.

## 4. Discussion

AP is one of the common causes of clinical acute abdomen, and most of the cases of AP are self-limiting. Approximately 15%–20% of patients with AP may worsen, may develop systemic inflammatory response syndrome, and then may develop multiple organ failure or local complications, including pancreatic necrosis, pseudocysts, or abscesses. Eventually, severe acute pancreatitis (SAP) can develop [29]. Studies have found that the excessive apoptosis of immune cells in SAP patients leads to immunosuppression, which is complicated by sepsis, septic shock, and multiple organ dysfunction syndrome (MODS), which are the main causes of late death [30]. An early and accurate assessment of the disease severity of AP is of great value for clinicians to formulate diagnosis and treatment plans and to improve the prognosis of patients.

In recent years, with the deepening of the research, scholars have developed a variety of scoring systems to evaluate the severity of AP, including the APACHE-II score [31], Ranson score [32], SIRS score, BISAP score, MCTSI score [33], SOFA score, qSOFA score, and so on. However, these scoring items have limitations, such as the presence of multiple factors that are evaluated, the complexity of the scoring systems, and the inability to dynamically monitor the scoring systems, and the operation of these scoring systems is cumbersome. Therefore, the lack of effective and simple tools to predict the prognosis of SAP are a major problem.

The combination of multiple indicators can improve the sensitivity or specificity of the diagnostic method, and a nomogram is a visual graph of the results of a multivariate regression analysis [34]. The advantage of the nomogram is that it transforms the complex regression equation into a visual graph, making the results of the regression analysis model more readable, intuitive, and simpler to evaluate for the patient. This is precisely because of the intuitive feature of the nomogram. It is cherished by many scholars in clinical medical research and is widely used as a visual statistical model to evaluate the prognosis and risk of diseases.

Biochemical tests are necessary for the admission of AP patients to the ICU, so these results are readily available. Recently, a series of diagnostic and prognostic markers alone and in combination have been evaluated in patients with AP [35–37]. Although numerous available biomarkers have been proposed for the prediction of the prognosis, there remains a lack of specific biomarkers for the early and reliable prediction of the AP severity.

Our study has established a visual nomogram for the early prediction of sepsis in patients with AP based on a large population obtained from critical illness databases, and we included variables such as albumin, lactate, the platelet count, phosphate, calcium, sodium, the Charlson Comorbidity Index (CCI), the GCS, temperature, urine output, and the MBP. These variables are easily available clinical indications and laboratory test indicators. It was previously confirmed that the above indicators are significantly related to SAP and its scoring system. The model has passed a series of validation and shows a predictive performance comparable to the current scoring system.

Previous studies have shown that the lactate/albumin ratio at admission can independently predict the mortality of patients with severe sepsis or septic shock. Our study found that albumin and lactate are independent risk factors for predicting a poor prognosis due to sepsis in AP patients in the ICU, and this is consistent with previous studies [38–40]. It has been reported that lactic acid is a reliable prognostic biomarker of multiple organ dysfunction and a poor health prognosis in patients with sepsis [41, 42]. In addition to lactic acid, the albumin level can reliably predict weakness, vulnerability to stress, and unstable homeostasis in patients and is related to the prognosis of severe diseases [43, 44]. Hypoalbuminemia is an indicator of an inflammatory state and can accurately predict the health outcomes of patients with chronic and inflammatory diseases [45, 46].

Platelets are small pieces of cytoplasm that are shed by mature megakaryocytes and are involved in the hemostatic function of the body. When the body develops stress effects secondary to acute and critical illness, the number of platelets will change, and the degree of platelet change will affect sepsis [47]. The severity of the disease has a certain relationship with the platelet level. A decrease in the number of platelets is an independent risk factor for critical illness. The action of inflammatory factors and intracellular toxin causes an increase in the negative hematopoietic regulators, an inhibition of the hematopoietic function of bone marrow, and a decrease in the number of mature megakaryocytes. At the same time, the number of platelets usually have a downward trend. Fever is a complex physiological reaction caused by pathogenic invasion. It can activate the immune response and increase the body temperature, and fever is the main symptom of an infection. An excessive rise in the body temperature can cause damage to tissues and organs, which can cause organ dysfunction and can cause metabolic disorders, especially when the body temperature is ≥39.5°C. When the body temperature is that high, the basal metabolic rate significantly increases, which worsens mitochondrial dysfunction and cellular ischemia and hypoxia, and fever can also accelerate glycolysis and reduce high energy expenditure. The production of phosphoric acid compounds causes an increase in the levels of lactic acid and can cause acidosis [48, 49], which increases the risk of a poor prognosis [50, 51]. Our study found that a low platelet count and significant hypothermia increase the risk of sepsis in patients with AP, which is consistent with the results of previous studies.

Electrolytes are an important part of various physiological processes and the maintenance of normal body functions. The electrolyte balance is regulated by hormone levels and the nutritional status of the patient. Electrolyte disorders are a reflection of pathological processes and are closely related to the development and prognosis of diseases. Serum phosphate disorders are common in critically ill patients. Phosphate in the form of triphosadenine is necessary for cell activity and is essential for improving the physiological functions of critically ill patients. Previous studies have confirmed that high phosphate is associated with an increase in the 28-day mortality in ICU patients [52]. And our findings are consistent with the previous studies. Previous studies have confirmed that decreased calcium ions in the serum are an indicator of a poor prognosis for pancreatitis patients [53]. ICU patients have a higher incidence of hypernatremia and have an increased in-hospital mortality [54–56]. Hypernatremia and hypocalcemia are caused by various pathological conditions in AP patients and increase the prognostic risk of sepsis, and our research has also confirmed this conclusion.

Urine output is part of the fluid output and is the main way for the body to excrete water. It is related to renal perfusion. Kidney damage and direct vasoconstriction of the renal blood vessels are the main reasons for decreases in urine output in SAP patients. The incidence of acute kidney injury in severe acute pancreatitis is between 14% and 43% [57], and the mortality rate is high. A decreased urine output indicates a poor prognosis for SAP patients. SAP patients often suffer from hypotension and shock, and when the MBP is too low, sepsis and septic shock symptoms are more likely to occur.

CCI is an assessment tool for predicting the risk of death due to disease by quantifying patient comorbidity. Numerous clinical studies have shown that the CCI has a strong ability to predict and judge a variety of diseases [58, 59]. The larger the CCI value, the more the comorbidities are present in AP patients, and the greater the risk of sepsis. Our study also found that the CCI is a positive predictor of a poor prognosis in AP patients. The GCS [60] was originally used as an assessment tool for patients with head injuries to assess the patient's coma. It has now become an important part of the system to determine the severity after an injury. Hietaranta [61] and other studies have confirmed that the early complications of severe acute pancreatitis and the occurrence of MODS are both closely related to SIRS or sepsis.

Our study has several strengths. This is the first study that aimed to propose a nomogram with easily obtainable laboratory measures. It provides a probability of a certain outcome (sepsis due to AP in our study) and allows practitioners to clearly interpret the prognosis of a patient using the individual nomogram estimates. The performance of our model is far superior to the traditional AP prognostic score.

Our research still has some limitations. Firstly, this is a retrospective analysis; therefore, the possibility of a selection bias cannot be ignored. Secondly, this study only included severe AP patients from a single center, which may prevent us from not extending the proposed nomogram to a larger population. Thirdly, insufficient information about the causative disease may lead to a bias in the multivariate analysis. Fourthly, because the data we based this study on are from an observational database, the results reported in our study should be regarded only as a reference and must be further verified externally.

## 5. Conclusion

Our model effectively predicts the risk of sepsis in AP patients in the ICU. Novel nomogram have high sensitivity and specificity and can be widely used if routine clinical data are available. The application of this nomogram in the clinical care environment can help clinicians make individual decisions in the treatment and management of these patients, thereby helping reduce the risk of sepsis and death in patients with AP.

## Figures and Tables

**Table 1:** Baseline characteristics and outcome of participants.

Variables	Total	Training set	Validation set	*P* value
*N*	1295	906	389	
Age	59 (47, 71)	58 (47, 70)	61 (47, 73)	0.110
Gender (%)				0.703
Male	766 (59.2)	539 (59.5)	227 (58.4)	
Female	529 (40.8)	367 (40.5)	162 (41.6)	
Ethnicity (%)				0.064
White	930 (71.8)	655 (72.3)	275 (70.7)	
Black	170 (13.1)	107 (11.8)	63 (16.2)	
Other	195 (15.1)	144 (15.9)	51 (13.1)	
Weight (kg)	81.3 (70,98.1)	81 (69,95.7)	82.1 (71.3,100)	0.083
PE (%)				0.545
No	1100 (84.9)	766 (84.5)	334 (85.9)	
Yes	195 (15.1)	140 (15.5)	55 (14.1)	
CCI	4 (2, 6)	4 (2, 6)	5 (2, 7)	0.341
GCS	15 (15, 15)	15 (15, 15)	15 (15, 15)	0.744
HR (min^−1^)	94.3 (81.3, 107.6)	94.8 (81.9, 107.6)	93 (79.5, 107.7)	0.341
MBP (mmHg)	80.2 (72.2, 90.7)	80.1 (71.8, 89.8)	80.5 (72.6, 91.9)	0.541
RR (min^−1^)	20.2 (17.3, 23.7)	20.2 (17.3, 23.6)	20.1 (17.2, 24)	0.796
Temperature (°C)	36.9 (36.7, 37.3)	36.9 (36.7, 37.3)	36.9 (36.7, 37.3)	0.647
Urine output (mL)	180 (75,300)	182.5 (75,300)	175 (65,325)	0.622
Spo2 (%)	96.4 (95, 97.9)	96.4 (95, 97.9)	96.5 (94.9, 97.8)	0.935
AG (mEq/L)	15 (13, 18)	15 (13, 18)	15 (13, 19)	0.759
Sodium (mEq/L)	138 (135,141)	138 (135,141)	139 (136,141)	0.067
Potassium (mEq/L)	4 (3.7, 4.5)	4 (3.6, 4.5)	4 (3.7, 4.5)	0.490
Magnesium (mEq/L)	1.8 (1.6, 2.1)	1.8 (1.6, 2.1)	1.8 (1.6, 2.1)	0.683
Calcium (mEq/L)	8.2 (7.5, 8.7)	8.2 (7.5, 8.7)	8.2 (7.6, 8.7)	0.477
Chloride (mEq/L)	104 (99, 108)	103.5 (99, 108)	104 (99, 108)	0.270
Phosphate (mEq/L)	3.3 (2.5, 4.1)	3.3 (2.5, 4.2)	3.4 (2.7, 4.1)	0.599
Bicarbonate (mEq/L)	22 (19, 25)	22 (19, 25)	23 (19, 25)	0.496
WBC (k/uL)	11.9 (7.8, 17)	12 (7.7, 17.1)	11.6 (7.9, 16.7)	0.355
RBC (m/uL)	3.7 (3.2, 4.3)	3.7 (3.2, 4.2)	3.8 (3.2, 4.4)	0.069
Platelet (k/uL)	197 (138, 280.5)	197 (135, 282)	199 (142, 276)	0.760
Hematocrit (%)	34.3 (29.6, 39.3)	34.2 (29.6, 39.1)	34.6 (30, 40)	0.137
MCHC (%)	33.1 (32, 34.1)	33.1 (32, 34.1)	33 (32, 34.1)	0.412
RDW (%)	14.6 (13.6, 16.1)	14.6 (13.7, 16.1)	14.5 (13.5, 16.2)	0.253
INR	1.3 (1.1, 1.5)	1.3 (1.1, 1.5)	1.3 (1.1, 1.5)	0.503
Creatinine (mg/dL)	1 (0.7, 1.7)	1 (0.7, 1.7)	1 (0.7, 1.5)	0.888
BUN (mg/dL)	20 (12, 33)	20 (12, 34)	20 (12, 33)	0.905
Lactate (mg/dL)	1.7 (1.2, 2.7)	1.7 (1.2, 2.7)	1.6 (1.2, 2.7)	0.782
Albumin (g/dL)	3 (2.6, 3.5)	3 (2.6, 3.6)	3 (2.6, 3.5)	0.473
Bilirubin (mg/dL)	0.9 (0.5, 2.5)	1 (0.5, 2.5)	0.9 (0.5, 2.2)	0.226
AP (IU/L)	100 (68,169)	99 (68,166)	101 (68,173)	0.945
AST (IU/L)	60 (31,161)	61 (31,160)	59 (30,161)	0.779
ALT (IU/L)	46 (22,127)	43 (21,123)	54 (25,141)	0.055
Glucose (mg/dL)	123 (101,168)	123 (100.2,169)	124 (102,162)	0.983
Lipase (IU/L)	201 (61,823)	196 (59.5,808.8)	220 (64,890)	0.422
LD (IU/dL)	320 (221.5, 483.5)	323 (221.2, 481)	316 (223,490)	0.804
BISAP	2 (1, 2)	2 (1, 2)	2 (1, 2)	0.384
SOFA	1 (0, 3)	1 (0, 3)	1 (0, 3)	0.358
qSOFA	1 (0, 1)	1 (0, 1)	1 (0, 1)	0.705
SIRS	3 (2, 3)	3 (2, 3)	3 (2, 4)	0.455
Sepsis (%)				0.943
No	554 (42.8)	387 (42.7)	167 (42.9)	
Yes	741 (57.2)	519 (57.3)	222 (57.1)	

Abbreviations: PE, pleural effusion; CCI, Charlson_comorbidity_index; GCS, Glasgow Coma Scale; HR, heart rate; MBP, mean blood pressure; RR, respiratory rate; SpO2, percutaneous oxygen saturation; AG, anion gap; WBC, white blood cell; RBC, red blood cell; MCHC, mean corpuscular hemoglobin concentration; RDW, RBC distribution width; INR, international normalized ratio; BUN, blood urea nitrogen; AP, alkaline phosphatase; AST, aspartate transaminase; ALT, alanine transaminase; LD, lactate dehydrogenase; BISAP, blood urea nitrogen, impaired mental status, systemic inflammatory response syndrome, age and pleural effusion; SOFA, Sequential Organ Failure Assessment; SIRS, systemic inflammatory response syndrome.

**Table 2:** Logistical analyses for patients with acute pancreatitis.

Variables	OR	95%CI	*P* value
MBP	0.975	0.964–0.987	<0.001
Temperature	1.876	1.437–2.449	<0.001
Urine output	0.999	0.998–1.000	0.005
GCS	0.86	0.763–0.969	0.013
CCI	1.098	1.035–1.164	0.002
Sodium	1.027	1.001–1.054	0.041
Calcium	0.841	0.730–0.970	0.017
Phosphate	1.264	1.134–1.409	<0.001
Platelet	0.999	0.998–1.000	0.017
Lactate	1.137	1.039–1.243	<0.001
Albumin	0.763	0.609–0.957	0.019

Abbreviations: MBP, mean blood pressure; GCS, Glasgow Coma Scale; CCI, Charlson_comorbidity_index; OR, odds ratio; CI, confidence interval.

**Table 3:** Predictive performances and validation of the nomogram.

Predictive model	AUC	*P* value	NRI	*P* value	IDI	*P* value
*Training cohort*						
Nomogram	0.730 (0.700–0.765)					
BISAP	0.632 (0.595–0.673)	<0.001	0.548 (0.379–0.736)	<0.001	0.118 (0.096–0.141)	<0.001
SOFA	0.627 (0.583–0.681)	<0.001	0.549 (0.423–0.721)	<0.001	0.124 (0.101–0.147)	<0.001
qSOFA	0.524 (0.481–0.562)	<0.001	0.726 (0.597–0.854)	<0.001	0.163 (0.139–0.187)	<0.001
SIRS	0.613 (0.573–0.652)	<0.001	0.550 (0.393–0.741)	<0.001	0.128 (0.102–0.154)	<0.001

*Validation cohort*						
Nomogram	0.732 (0.680–0.780)					
BISAP	0.666 (0.609–0.723)	0.005	0.460 (0.165–0.751)	<0.001	0.092 (0.056–0.128)	<0.001
SOFA	0.677 (0.595–0.747)	<0.001	0.588 (0.375–0.863)	<0.001	0.133 (0.098–0.168)	<0.001
qSOFA	0.488 (0.421–0.549)	<0.001	0.718 (0.552–0.947)	<0.001	0.168 (0.131–0.204)	<0.001
SIRS	0.614 (0.552–0.673)	<0.001	0.592 (0.370–0.865)	<0.001	0.136 (0.097–0.175)	<0.001

Abbreviations: AUC, area under the receiver operating characteristic curve; NRI, net reclassification improvement; IDI, integrated discrimination improvement; BISAP, blood urea nitrogen, impaired mental status, systemic inflammatory response syndrome, age and pleural effusion; SOFA, Sequential Organ Failure Assessment; SIRS, systemic inflammatory response syndrome.

## Data Availability

The MIMIC-IV data are available on the project website at https://mimic-iv.mit.edu/. But the validation set generated for this article is not readily available because the ethics committee does not allow the release of the data.
